# Synchrotron and Raman Study of the Rotator Phases and Polymorphism in Tricosane Paraffin

**DOI:** 10.3390/polym12061341

**Published:** 2020-06-13

**Authors:** Enrique Blázquez-Blázquez, Rosa Barranco-García, María L. Cerrada, Juan C. Martínez, Ernesto Pérez

**Affiliations:** 1 Instituto de Ciencia y Tecnología de Polímeros (ICTP-CSIC), Juan de la Cierva 3, 28006 Madrid, Spain; enrique.blazquez@ictp.csic.es (E.B.-B.); rbarranco@ictp.csic.es (R.B.-G.); mlcerrada@ictp.csic.es (M.L.C.); 2 ALBA Synchrotron Light Source, Carrer de la Llum 2-26, Cerdanyola del Vallès, 08290 Barcelona, Spain; guilmar@cells.es

**Keywords:** tricosane paraffin, synchrotron SAXS and WAXS, polymorphism, rotator phases, variable-temperature Raman spectroscopy, transient structures

## Abstract

A detailed study of the phase behavior of *n*-paraffin C_23_H_48_ has been performed by means of real-time variable-temperature experiments with synchrotron radiation. Two detectors were employed for simultaneous analysis of the small-angle (SAXS) and wide-angle X-ray-scattering (WAXS) regions. This paraffin presents a very interesting phase behavior, involving two crystal polymorphs, three rotator phases and the liquid state. The Ostwald rule of stages is invoked to find similarities of the rotator phases with the eventual transient mesomorphic structure in the multistage model of polymer crystallization. That study is complemented by variable-temperature Raman experiments covering frequencies down to 150 cm^−1^. It was found that the low-frequency region is the most informative regarding the phase transitions, and specifically the intensity of the first overtone. From these analyses, several parameters are evaluated as function of temperature.

## 1. Introduction

Many studies have been devoted to the phase behavior of *n*-paraffins. This considerable interest has been partly due to the fact that these simple compounds can be considered as models for the structure of many other more complicated systems in different fields, such as liquid crystals, surfactants, oils, membranes, lipids and other biological compounds [1,2,3,4,5,6,7] with properties highly dependent on the aliphatic component. Consequently, any information related to the phase behavior of the constituent paraffins will be of significance for a better understanding of the performance of those materials, most of them with paramount industrial relevance.

Furthermore, there are additional implications in the crystalline structure of a polymer as relevant as polyethylene, which can be considered as the paraffin with sufficiently high *n*.

These n-paraffins exhibit a rather complicated and attractive phase behavior, showing different crystalline polymorphs depending on the number of carbons, on the parity, and, of course, on temperature [8,9]. Besides, different solid–solid phase transformations are observed, especially for homologues with *n* > 22.

Furthermore, and since the pioneer work of Muller [10], it was realized that between those polymorphs with perfect crystalline order and the fully disordered liquid phase, there are the so-called rotator phases. These phases exhibit a typically crystalline long-range three-dimensional positional order, but accompanied by a rotational freedom of the molecules about its long axis [1,2]. The result is a layered organization with a certain disorder, so that these phases are also named as plastic crystals, resembling the structure of highly ordered smectics.

In recent years, there has been also a substantial attention to transient structures in polymer crystallization with the underlying idea that the final crystal is attained via a phase (mesophase) intermediate between the disordered amorphous and the three-dimensional crystal. It has to be considered that, at the nanometric level, the kinetic aspects may be more relevant than the relative stability of the phases, as stated by the Ostwald rule of stages [11]. As a result, the mesophase will be eventually the initial step of the polymer crystallization process [12,13,14,15,16,17,18,19], and, for instance, the crystallization of polyethylene might proceed via such intermediate phase, which may be similar in certain aspects to the rotator phases found in *n*-paraffins.

Up to five different rotator phases have been described in *n*-paraffins [1,2,3,4,20,21,22,23,24,25,26,27,28,29], usually named as R-I to R-V, the order number coming from their reporting date rather than the location in the eventual phase diagrams.

In the R-I phase, the molecules are untilted in relation to the layers, and they form a hexagonal lattice that is rectangularly distorted, with a bilayer stacking. This phase is also named as face-centered orthorhombic.

The R-II rotator phase (also named as rhombohedral) is the one of highest symmetry, and also comprises molecules that are untilted in relation to the layers, being organized in a hexagonal packing, but now with a trilayer sequence. It resembles the liquid-crystalline smectic-B phase [20,21].

These two phases were initially called low-temperature and high-temperature rotator phases, since the phase sequence found in some *n*-paraffins of short chains is, on heating: crystal → R-I → R-II → liquid.

The rotator phases R-III and R-IV are both present tilted molecules in relation to layers, the first one with a triclinic structure, while the second is ordered into a monoclinic arrangement.

Finally, R-V shows a base-centered monoclinic structure with a bilayer stacking and tilted molecules.

The phase behavior of odd paraffins between C9 and C21 is relatively simple, since only one rotator phase is observed before the final transition to the liquid state [22,23]. The situation is different for longer homologues, where several rotator phases appear. This is the case for tricosane, C_23_H_48_, since three different rotator phases have been reported [1,2,4,22,23,28,29].

Obviously, X-ray diffraction is the most appropriate technique for studying the phase behavior in general. Nevertheless, Raman spectroscopy is also a suitable tool for that purpose, considering that it is sensitive to conformational changes [30]. Therefore, transitions among either the different crystalline polymorphs, or between crystal and rotator phases, and also between rotator phases and the melt can be studied by this technique [6].

Focusing the attention on paraffin C_23_H_48_ (abbreviated as C23), most of the reported studies using X-ray diffraction do not present a systematic analysis of the variation in temperature. In the case of Raman spectroscopy, several reports have been devoted to this paraffin C23, including the analysis at different pressures [31,32,33], but in general there are a lack of experimental data at low frequencies, below around 600 cm^−1^, where the accordion modes and their overtones are observed.

We present here a detailed study of the phase behavior of C23 by means of real-time variable-temperature experiments with synchrotron radiation, by simultaneously analyzing the small-angle (SAXS) and wide-angle X-ray scattering (WAXS) regions. The use of the SAXS detector can obtain the layer spacings with high accuracy. This study is complemented by variable-temperature Raman experiments covering frequencies down to 150 cm^−1^. From these analyses, several parameters are evaluated as function of temperature in this paraffin, which shows a very interesting phase behavior, involving two crystal polymorphs, three rotator phases and the liquid state.

## 2. Materials and Methods

High-purity tricosane (Aldrich, 99% nominal) was used in this study. The sample has been analyzed by gas chromatography-mass spectroscopy (GC-MS) in a Hewlett Packard 6890 GC gas chromatograph provided with a mass spectrometry detector model 5973 (Agilent Technologies) and a DB5-HT capillary column (15 m length × 250 μm internal diameter and 0.1 μm film thickness). Helium was used as carrier gas (flow rate = 1 mL/min). The electron impact (70 eV) was the selected type of ionization for the mass spectrometer.

Differential scanning calorimetry (DSC) experiments were recorded in a TA Instruments Q100 calorimeter (provided with a cooling system and calibrated with different standards). Well-sealed liquid sample pans were used.

Real-time variable-temperature SAXS/WAXS experiments with synchrotron radiation were performed at beamline BL11-NCD-SWEET at ALBA (Cerdanyola del Vallés, Barcelona, Spain) at a fixed wavelength of 0.1 nm. The WAXS profiles were acquired with a Rayonix LX255-HS detector, placed at about 16 cm from sample and a tilt angle of around 30 degrees, and the SAXS ones with a Pilatus 1M detector (at a distance of around 300 cm from sample). The temperature control unit was a Linkam hot stage, connected to a cooling system working with liquid nitrogen. The calibration of spacings was obtained by means of silver behenate and Cr_2_O_3_ standards. The initial 2D X-ray images were converted into 1D diffractograms, as function of the inverse scattering vector, *s* = 1/*d* = 2 sin θ/*λ*, by means of pyFAI python code (ESRF) [34], modified by ALBA beamline staff.

A scanning rate of 2 °C/min was used on these experiments. Since the scattering patterns were collected in time frames of 3 s, it follows that there is a temperature difference of 0.1 °C between consecutive frames. For practical purposes, diffractograms were acquired in the temperature interval from 36.0 to 52.0 °C.

Raman experiments were performed in the Raman Micro-spectroscopy Laboratory of the Characterization Service at ICTP-CSIC, by using a Renishaw InVia Reflex Raman system (Renishaw plc, Wotton-under-Edge, UK), provided with a grating spectrometer, coupled to a confocal microscope, and with a Peltier-cooled charge-coupled device (CCD) detector. A diode laser (wavelength of 785 nm) is used for exciting the Raman scattering. The laser beam, with a power of 160 mW, is focused at the sample with the aid of a 0.85 × 100 microscope objective. An exposure time of 1 s and 200 accumulations were employed in these Raman measurements.

The temperature control was provided by a Linkam temperature unit (THMS 600 model) coupled to the system. Taking into account that the total acquisition time for a spectrum is much higher than that for the diffractograms in the synchrotron experiments, a step-temperature method was used for these Raman experiments, by increasing the temperature 0.5 °C and acquiring the spectra.

## 3. Results and Discussion

**Figure 1:**
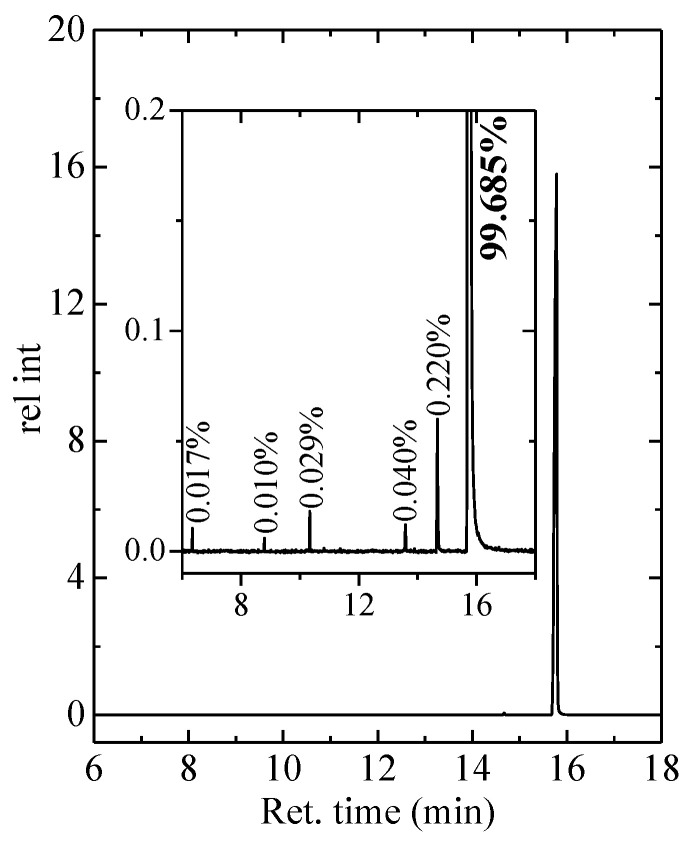
Gas chromatography mass spectrometry (GC-MS) chromatogram of C_23_H_48_.

Figure 1 shows the GC-MS chromatogram of the C23 sample. The purity deduced from this analysis is as high as around 99.7% for the sample under study. Knowledge of purity is important because of the notorious effects of impurities on the phase transitions [29].

**Figure 2:**
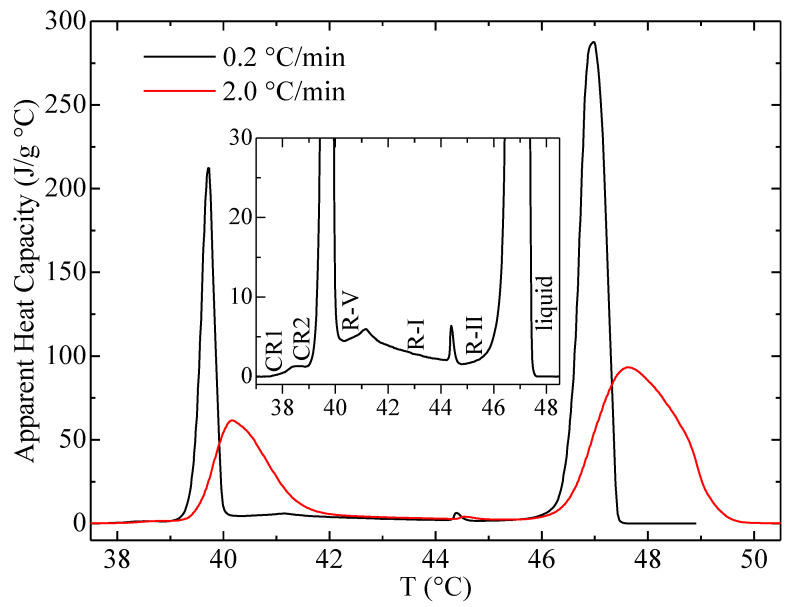
Differential scanning calorimetry (DSC) heating curves, at the indicated scanning rates, for the C_23_H_48_ sample.

The DSC-heating curves of the C23 sample are shown in Figure 2. As observed, two different scanning rates have been used: one of them is 2 °C/min, which is that employed in the synchrotron experiments. The other scanning rate is 0.2 °C/min, chosen in order to have a much better resolution, especially for those transitions involving a low-heat-capacity change. These transitions are clearly noticeable in the amplified inset.

Thus, five transitions take place for C23, at the following onset temperatures: 38.0, 39.4, 40.8, 44.3 and 46.5 °C. These temperatures agree rather well with the values reported before (different values collected in [29]). The nature of these transitions has been reported [2,4,22,29] to be that shown in the inset of Figure 2, and it will be ascertained and explained below, in light of the synchrotron experiments. It is important to mention that the considerable overheating displayed in the DSC curve at 2 °C/min means that the transitions with onsets at 39.4 and 40.8 °C appear merged in a single peak.

**Figure 3:**
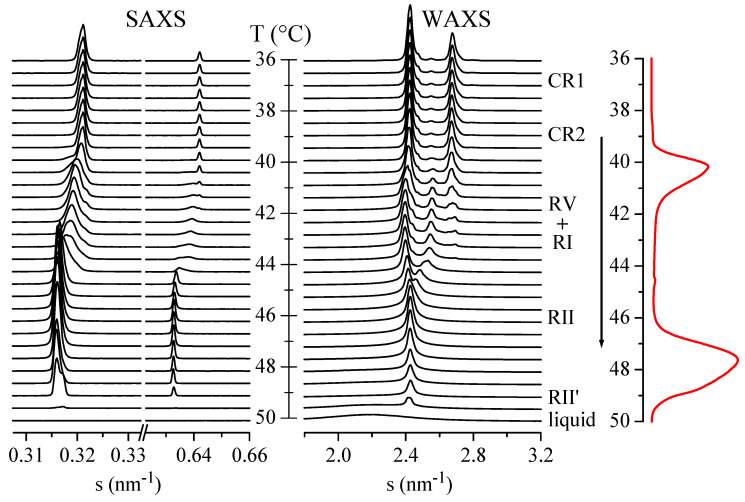
Small-angle X-ray scattering (SAXS)/wide-angle (WA)-XS diffractograms for sample C_23_H_48_ in a heating experiment at 2 °C/min. The corresponding DSC heating curve is also shown at the right. For clarity of the presentation, only one out of every five diffractograms is plotted.

As commented above, real-time variable-temperature SAXS/WAXS experiments with synchrotron radiation were performed in the temperature interval from 36.0 to 52.0 °C, which covers all the aforementioned transitions. Figure 3 shows these SAXS/WAXS diffractograms for sample C23 in a heating experiment at 2 °C/min. It can be observed that there is no appreciable change up to around 39.5 °C, which includes the first transition mentioned above with a DSC onset of 38.0 °C. Therefore, the phases involved in that transition have to be structurally rather similar.

Above 39.5 °C, both the SAXS and WAXS profiles change considerably, coinciding with the main DSC transition with onset at 39.4 °C. Thus, Figure 3 shows that the main SAXS peak and its second order (and other orders not shown in the figure) undergo a continuous shift to lower *s* values (higher *d* spacings). These features are characteristic of the formation of the rotator phase R-V, which is subsequently transformed into the R-I rotator phase. As mentioned above, the two transitions with onsets at 39.4 and 40.8 °C are merged in what seems to be a single peak at this heating rate of 2 °C/min.

The next transition occurs at around 44.5 °C, corresponding with the appearance of the rhombohedral R-II rotator phase. This begins to melt at about 46 °C, which is characterized in the WAXS diffractograms by the formation of a broad peak centered at around *s* = 2.18 nm^−1^ (*d* = 0.46 nm). This broad peak increases its area in relation to that of the R-II phase, until its total melting at 50 °C. The liquid phase is characterized by such broad WAXS peak and the absence of any layer reflection in the SAXS channels (see lower diffractograms in Figure 3).

Interestingly, there is a small shift in the SAXS peak above around 49 °C, yet keeping the characteristic peak of a rhombohedral phase in WAXS. It seems, therefore, that a new rotator phase very similar to R-II is formed. Thus, we will tentatively call it the R-II′ phase. In fact, the DSC melting peak centered at around 48 °C shows a kind of high-temperature component, which may be associated with that phase change.

It has been reported for several kinds of ethylene copolymers [35,36,37,38,39,40,41] with high comonomer contents that, in addition to the usual (110) and (200) reflections of orthorhombic polyethylene, there was a third crystalline reflection, centered at around 0.453 nm. This reflection was tentatively assigned to hexagonal crystals, which in fact are rather imperfect and disordered [42,43], and possibly with a bundle-like arrangement and not the usual lamellar one. Moreover, a kind of rotator hexagonal phase [44] (or pseudo-hexagonal [45]) was also reported for ethylene-propylene copolymers at around 15–20 mol % in propylene.

In addition, a transient hexagonal phase before melting the orthorhombic crystals has been also reported for polyethylene at high pressure and temperature [46].

In the case of short *n*-paraffins, the molecules can rotate independently (even though there is not a prevailing conformational disorder) and this rotator phase is found to be a precursor of subsequent crystallization. However, for polymers (or long *n*-paraffins) the claimed hexagonal rotator phase mostly arises from conformational disorder.

This hexagonal phase would be similar to the transient mesomorphic structure invoked in the multistage model of polymer crystallization [15]. As stated before [18], evidence for the crystallization of polyethylene via such transient structures are circumstantial, although suggestive. In any case, the granular structures sometimes observed in polymeric crystals have been proposed to be a fingerprint of those transient states [47].

Now, a more detailed analysis is carried out for some parameters related to all those phase transitions in C23.

As mentioned above, there is no appreciable change in the diffractograms up to around 39.5 °C, which includes the first DSC transition with an onset of 38.0 °C. This endotherm has been found for the odd paraffins C23, C25, C27 and C29, being assigned to the so called δ transition [22,28,29,48], involving the change from two orthorhombic crystal structures (*Pbcm* to *Pbnm*). These two crystal phases will be designated as CR1 and CR2, respectively, in the following.

It has been reported that neither the position nor intensity of the different X-ray diffractions show appreciable variations through this transition [22], although another study [2] indicated that there is a small modification in the layer spacing, ranging from 3.109 nm for CR1 to 3.120 nm for CR2.

**Figure 4:**
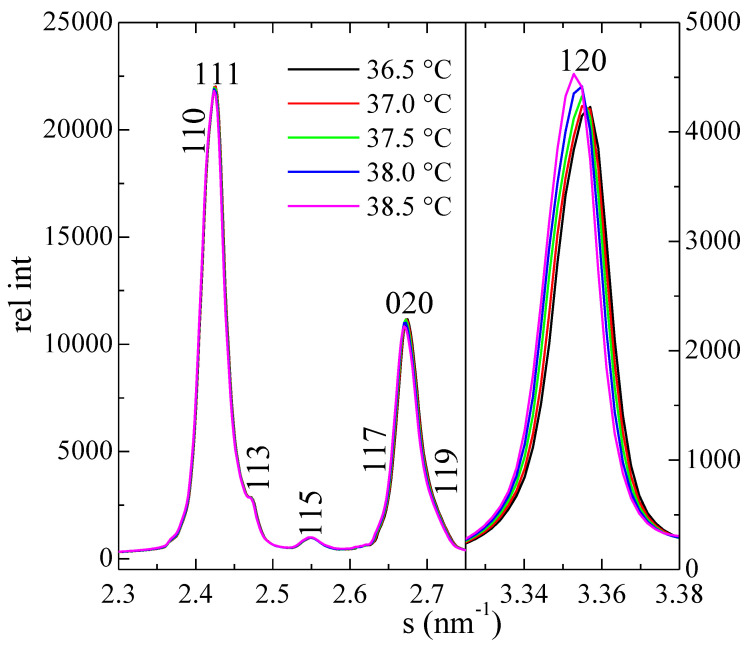
Selected regions of the WAXS diffractograms for sample C_23_H_48_ at the indicated temperatures covering the δ transition. The Miller indices are specified (note that *a* and *b* axes are interchanged in relation to polyethylene).

**Figure 5:**
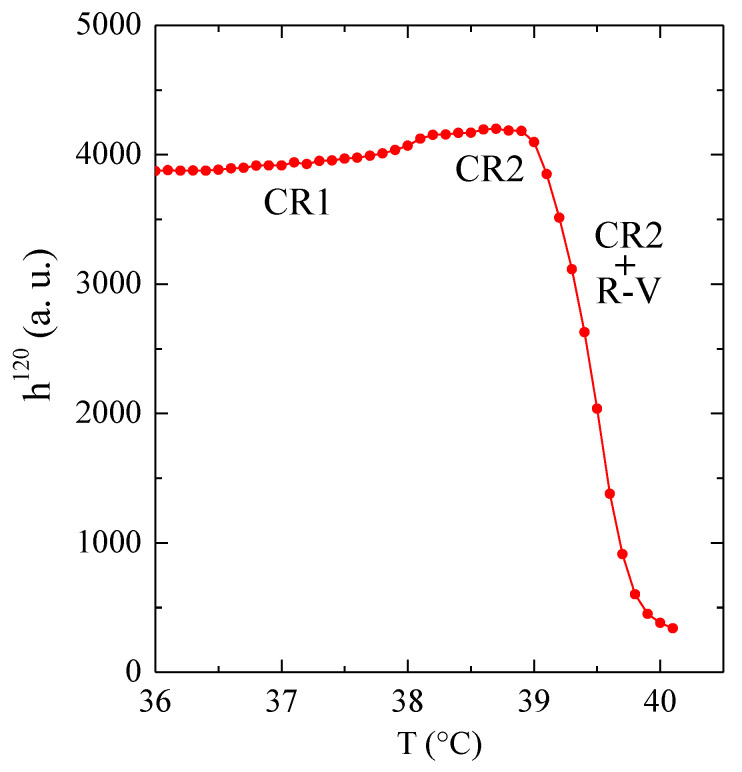
Dependence on temperature of peak height for the diffraction (120) in the vicinity of the δ transition (from CR1 to CR2) for sample C_23_H_48_.

Our results for sample C23 in the temperature interval of this δ transition are represented in Figure 4 in selected regions of the WAXS diffractograms. They indicate that there is no specific change (besides the logical small expansion with temperature) in most of the diffractions, excepting the (120) one, which shows a certain change in intensity, as displayed in Figure 4. A discontinuity is clearly noticeable in Figure 5, which shows the variation in the temperature of peak height for the diffraction (120). The δ transition appears characterized by a small step at 38 °C. At a slightly higher temperature, the transition to the R-V phase is much more clearly noticed in the intensity of that diffraction, which disappears totally at around 41 °C, when the transition into the R-V phase is complete.

**Figure 6:**
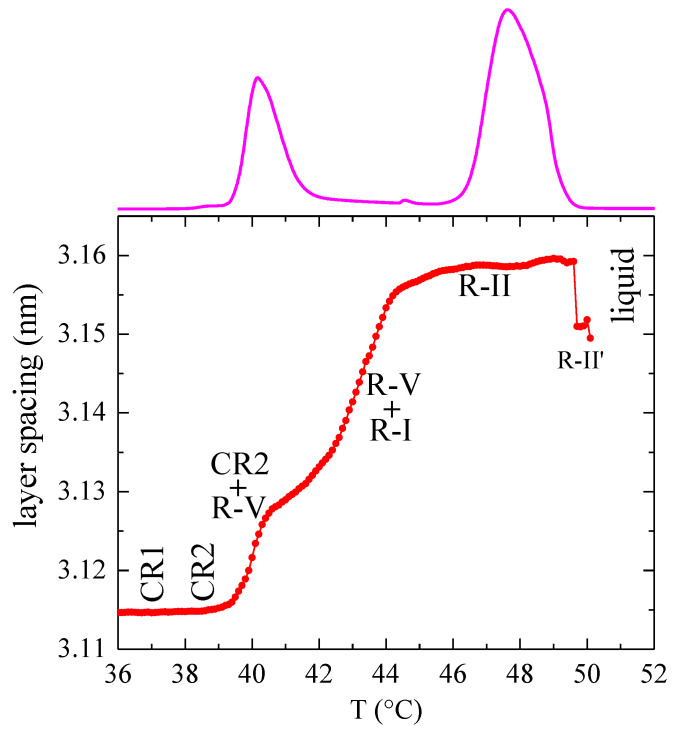
Dependence on temperature of the layer spacing for sample C_23_H_48_. The corresponding DSC heating curve is also shown at the top.

Another important aspect is the dependence of the layer spacing on temperature in the entire experiment. That variation, displayed in Figure 6, is estimated by changes in the spacing corresponding to the SAXS peak maximum. It means that, in the case of peak splitting due to the occurrence of simultaneous phases, the values depicted in Figure 6 will represent the component with the highest intensity. The first aspect deduced from this figure is the lack of appreciable variation in the layer spacing through the δ transition (which takes place at 38 °C, as deduced from Figure 5).

Secondly, and as commented above, the two transitions CR2 to R-V and R-V to R-I are merged at the heating rate of 2 °C/min in what appears to be a single DSC peak. This means that probably the R-V phase is always accompanied by a certain proportion of either CR2 or R-I [2], and thus the layer spacing shows a continuous but irregular increase from around 39 to 46 °C. At higher temperatures, the layer spacing levels off for the R-II phase. Above 47 °C, the latter phase will be accompanied with a certain proportion of liquid, but that is obviously not reflected in the variation in the spacing, since the liquid phase does not present any SAXS spacing.

Finally, there is a decrease in the layer spacing at the end of the melting peak, which is ascribed, as mentioned above, to the formation of a R-II′ phase. This possible new phase appears to have a geometry rather similar to that exhibited by RII, and one could think that it is just a consequence of the melting process, reflecting a departure of the *all-trans* conformation. However, if this were the case, a continuous decrease in the layer spacing would occur, while the experimental results in Figure 6 show a clear step.

The layer spacings shown in Figure 6 are in fairly good agreement with those reported before [2], excepting the ones for the tentative R-II′ phase, which were not described until the present work.

**Figure 7:**
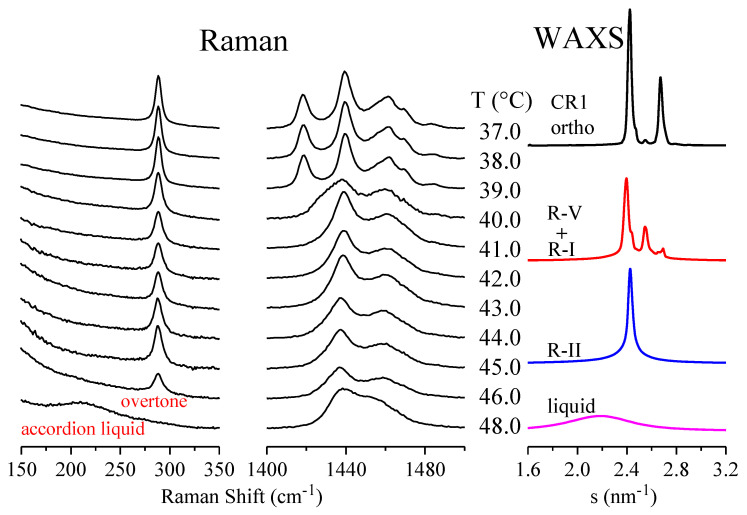
Selected sections of the Raman spectra for sample C_23_H_48_ in a step-heating experiment. Left: first overtone (and accordion liquid); middle: CH_2_ bend region. For clarity of the presentation, only one out of every two spectra is plotted. The corresponding WAXS diffractograms are also shown at the right.

As mentioned above, Raman spectroscopy is also a very suitable tool for the study of phase transitions involving either crystalline or rotator phases, and even the liquid state. Figure 7 shows selected representative sections of the Raman spectra for sample C23 in a step-heating experiment (see experimental): the low-frequency region and the CH_2_ bend region (the corresponding WAXS diffractograms are also shown at the right of the figure). Those two regions have been chosen since they are the most informative for the analysis of the different transitions.

Unfortunately, the lower limit of the Raman equipment is 150 cm^−1^, i.e., the accordion mode of C23 cannot be seen, since it is expected [32,49,50,51] to appear at around 105 cm^−1^. We are limited, therefore, to analyzing the first overtone, which is plotted in the left part of Figure 7 as a function of temperature. It can be observed that its position practically does not change, but the intensity is noticeably dependent on the phase involved, with a clear decrease for the rotator phases. Anyway, these overtones display rather similar characteristics when passing from the crystal to the rotator phases, as previously noticed for C19 [30].

These rotator phases involve chains that are mainly in trans conformation, but they are able to rotate along their chain axes, and, consequently, they exhibit Raman bands with rather narrow widths, similarly to the crystal. On the contrary, the disordered liquid phase comprises a high proportion of gauche conformers, leading to broader (and asymmetric) bands [49,52].

It is important to note, however, that the significant proportion of gauche conformers in the liquid leads to an important positive shift in the frequency of the accordion modes, and, notably, the variation in the number of carbons is considerably smaller than that for the crystal. Thus, a variation from 214 cm^−1^ in C17 to 209 cm^−1^ in C24 has been reported [53]. In the present case of C23, it can be observed in the lower spectrum of Figure 7, represented on the left, that there is a broad asymmetric band centered at around 214 cm^−1^, which is, therefore, assigned to the accordion band of the liquid.

Concerning the CH_2_ bend region, the band at 1418 cm^−1^ is characteristic of the orthorhombic crystal packing with *all trans* conformation, and it is also observed in polyethylene [54] and its copolymers [37,38]. In the present case of C23 (middle plots in Figure 7), this band is present at the lower temperatures (the crystal) but disappears above around 40.5 °C, being completely absent, therefore, for all the rotator phases (and for the liquid).

**Figure 8:**
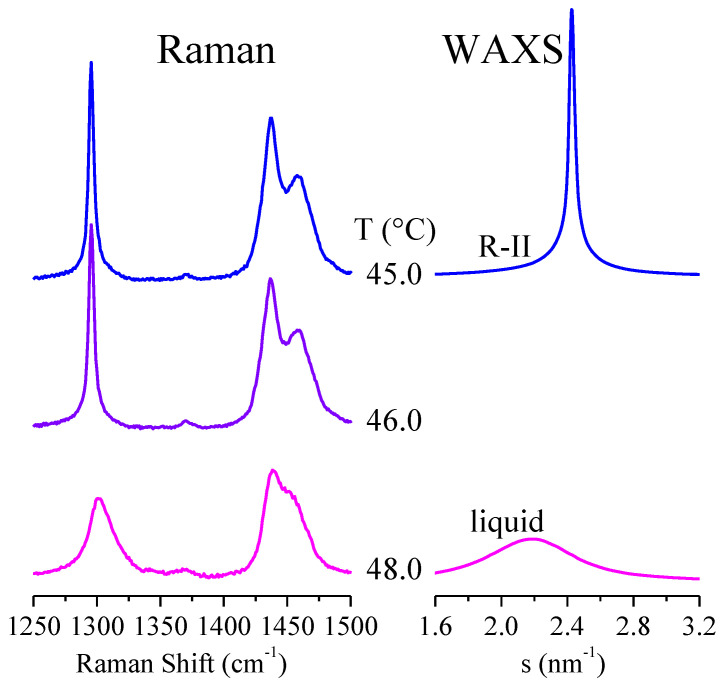
The CH_2_ twist and CH_2_ bend regions of the Raman spectra for sample C_23_H_48_ in the transition from the rhombohedral R-II rotator phase to the liquid state. The corresponding WAXS diffractograms are also shown at the right.

The broadening in the bands for the liquid is also clearly observed in Figure 8, particularly in the CH_2_ twist region during the transition from the rhombohedral R-II rotator phase to the liquid state. The narrow band centered at 1295 cm^−1^ for the rotator phase becomes a broader peak for the liquid, and its location undergoes also a shift of up to 1303 cm^−1^.

**Figure 9:**
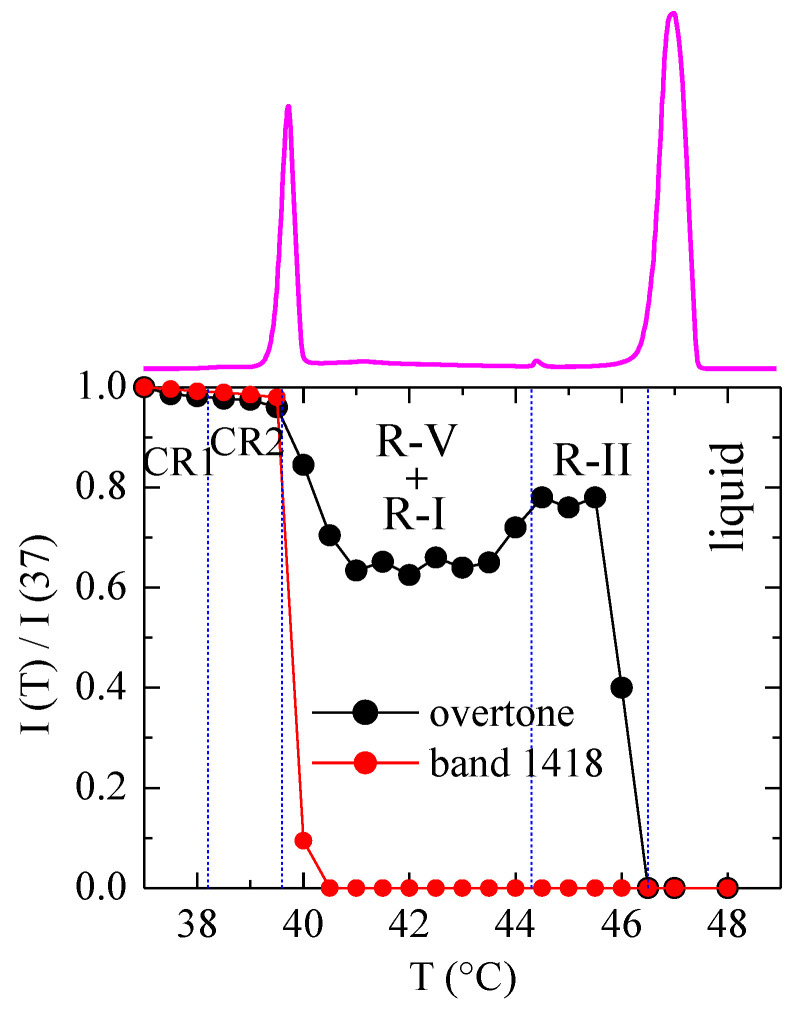
Variation in temperature of the ratio of intensity at temperature T to that at 37 °C for the first overtone and for the band at 1418 cm^−1^. The corresponding DSC heating curve is also shown at the top.

The next step addresses the analysis as function of temperature (and, implicitly, with the phase present) of some parameters related to these Raman spectra. Thus, Figure 9 shows such variation for the ratio of the intensity at temperature T to that at 37 °C for the first overtone and for the band at 1418 cm^−1^. Since these Raman experiments (see Experimental) have been acquired in a step-temperature mode (with an average heating rate smaller than 0.2 °C/min), the DSC heating curve shown at the top of Figure 9 corresponds to a heating rate of 0.2 °C/min (see Figure 2).

As mentioned, the band at 1418 cm^−1^ disappears above around 40.5 °C, so that the ratio I(T)/I(37) for this band is zero for all the rotator phases (and for the liquid). More informative is the ratio for the overtone. As observed, it decreases very slightly (temperature effect only) until 40 °C, with no appreciable discontinuity through the transition from CR1 to CR2. Next, it decreases by around 40% when reaching the R-V and R-I phases, and, interestingly, there is a significant increase when R-II is formed. The intensity of this first overtone (and probably also of the accordion mode, not seen here) is, therefore, rather dependent on the phase present.

**Figure 10:**
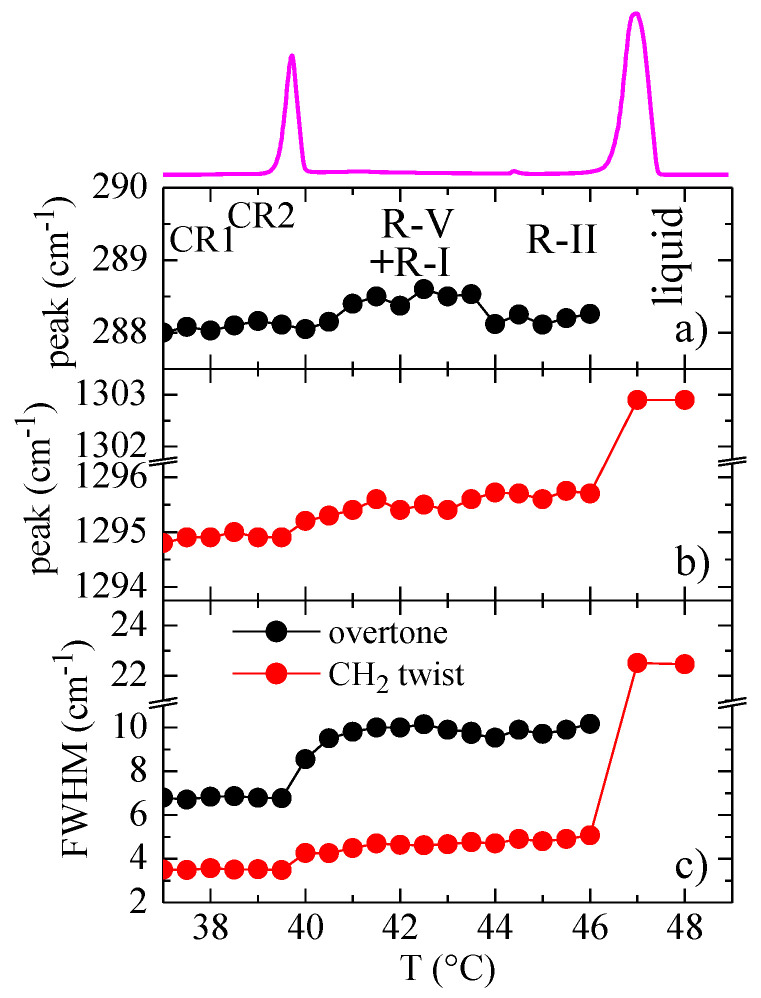
Dependence on temperature of: (**a**) the peak position of the first overtone; (**b**) the peak position of the CH_2_ twist band, and (**c**) the full width at half maximum of those two bands. The corresponding DSC heating curve is also shown at the top.

Figure 10a shows the dependence on temperature of the peak position of the first overtone. It displays a subtle increase, almost inside the experimental error, when R-V and R-I are present, but decreases again to the initial values for R-II. The peak position of the CH_2_ twist band (Figure 10b) shows also a very minor increase when passing from the crystal to the rotator phases, keeping practically constant through the three rotator phases, and an important rise from around 1295 to 1303 cm^−1^ for the liquid.

The variation of the full width at half maximum, FWHM, of those two bands (Figure 10c) is also clearly sensitive to the crystal–rotator transition, especially the one for the overtone, but there is not a clear dependence of FWHM with the kind of rotator phase. The increase is much more noticeable for the width of the CH_2_ twist band during the transition to the liquid, where the FWHM is more than five times greater than the one either for the rotator phase or for the crystal.

In summary, some variations are observed in several bands of the Raman spectra of C23 depending on the phase present, but the most informative region is the one at low frequencies, which was not systematically analyzed in previous reports.

## 4. Conclusions

A detailed study of the phase behavior of *n*-paraffin C_23_H_48_ has been performed by means of real-time variable-temperature experiments with synchrotron radiation, complemented by variable-temperature Raman experiments covering frequencies down to 150 cm^−1^.

This paraffin presents a very interesting phase behavior, involving two crystal polymorphs (CR1 and CR2), three rotator phases (R-V, R-I and R-II) and the liquid. The present synchrotron study, by simultaneously analyzing the small-angle (SAXS) and wide-angle X-ray scattering (WAXS) regions, is in accordance with that phase behavior, previously reported.

The use of the SAXS detector can obtain the layer spacing with high accuracy. It was found that layer spacing is not sensitive to the transition from CR1 to CR2. Only a very subtle discontinuity at around 38 °C in the intensity of WAXS diffraction (120) characterizes this crystal–crystal transition.

At higher temperatures, the layer spacing shows a continuous but irregular increase from around 39 to 44 °C, interpreted by considering that probably the R-V phase is always accompanied by a certain proportion of either CR2 or R-I.

Above 45 °C, the layer spacing levels off for the R-II phase, accompanied by a certain proportion of liquid beyond 47 °C. Finally, at the end of the final melting peak there is a decrease in the layer spacing, tentatively ascribed to the formation of a new rhombohedral R-II′ phase.

Some similarities between the rotator phases with the eventual transient mesomorphic structure in the multistage model of polymer crystallization are suggested, by invoking the Ostwald rule of stages.

The variable-temperature Raman experiments indicate that the low-frequency region (not addressed in previous reports) is the most informative regarding the phase transitions. Specifically, the intensity of the first overtone does not show appreciable discontinuity through the CR1-CR2 transition, but it decreases by around 40% when reaching the R-V and R-I phases. Interestingly, there is an additional significant increase when R-II is formed. The intensity of this first overtone (and probably also of the accordion mode, not seen under the present experimental conditions) is, therefore, rather dependent on the phase present.

Variation with temperature of the FWHM for the first overtone and for the CH_2_ twist band is also clearly sensitive to the crystal–rotator transition, especially the one for the overtone, but there is no clear dependence of the FWHM on the kind of rotator phase.
